# A Bayesian Filtering Approach for Error Mitigation in Ultra-Wideband Ranging

**DOI:** 10.3390/s19030440

**Published:** 2019-01-22

**Authors:** Jing Xin, Kaiyuan Gao, Mao Shan, Bo Yan, Ding Liu

**Affiliations:** 1 Shaanxi Key Laboratory of Complex System Control and Intelligent Information Processing, Xi’an University of Technology, Xi’an 710048, China; xinj@xaut.edu.cn (J.X.); 15002926726@163.com (K.G.); 18691263953@163.com (B.Y.); liud@xaut.edu.cn (D.L.); 2 Australian Centre for Field Robotics, The University of Sydney, Sydney, NSW 2006, Australia

**Keywords:** UWB ranging, NLOS, error mitigation, probabilistic sensor model, Bayesian filtering

## Abstract

Ultra-wideband (UWB) sensors have been widely used in multi-robot systems for cooperative tracking and positioning purposes due to their advantages such as high ranging accuracy and good real-time performance. In order to reduce the influence of non-line-of-sight (NLOS) UWB communication caused by the presence of obstacles on ranging accuracy in indoor environments, the paper proposes a novel Bayesian filtering approach for UWB ranging error mitigation. Nonparametric UWB sensor models, namely received signal strength (RSS) model and time of arrival (TOA) model, are constructed to capture the probabilistic noise characteristics under the influence of different obstruction conditions and materials within a typical indoor environment. The proposed Bayesian filtering approach can be used either as a standalone error mitigation approach for peer-to-peer (P2P) ranging, or as a part of a higher level Bayesian state estimation framework. Experiments were conducted to validate and evaluate the proposed approach in two configurations, i.e., inter-robot ranging, and mobile robot tracking in a wireless sensor network. The experimental results show that the proposed method can accurately identify the line-of-sight (LOS) and NLOS scenarios with wood and metal obstacles in a probabilistic representation and effectively improve the ranging/tracking accuracy. In addition, the low computational overhead of the approach makes it attractive in real-time systems.

## 1. Introduction

Tracking and positioning is a key problem in a variety of automation and robotics fields. They are considered a prerequisite for robots to conduct many tasks such as path planning and making behavioural decisions. In a multi-robot system, it is essential for each robot to determine positions of itself as well as its companions so as to improve efficiency and safety when they are collaborating together [1]. The global navigation satellite system (GNSS) based positioning has been extensively used in outdoor environments, with positioning accuracy in the magnitude of 1 to 10 m. When it comes to indoor positioning/tracking, GNSS is known to suffer from significant attenuation of satellite signal when penetrating obstacles such as building roofs and walls [2]. In addition, the indoor positioning problem poses a serious challenge of requiring a higher level of accuracy than current GNSS technology can offer. A common way for indoor positioning is to distribute multiple anchor/reference points with static and predetermined positions in the environment, and the position of a mobile robot, defined as a tag point here, can be determined using triangulation or trilateration within the wireless sensor network. The relative inter-robot ranging information can be further fused into a cooperative tracking framework to determine coordinates of multiple mobile robots [3].

Widely used wireless ranging techniques can be mainly categorized into ultrasonic based, Bluetooth based, radio-frequency identification (RFID) based, WiFi based, and ultra-wideband (UWB) based approaches. Compared with the rest of wireless ranging techniques, UWB based methods are favored because of its high ranging accuracy, good real-time performance, and strong signal penetration capability [4]. It gradually has become the preferred technology for high-precision localization/tracking in indoor and structured environments. Four typical range based positioning algorithms, namely analytical, least squares, Taylor series, and extended Kalman filter, are studied in [5]. A statistical model is proposed in [6] for mitigating ranging error only in line-of-sight (*LOS*) propagation scenario. Ref. [7] investigates the scalability of indoor localization solutions and demonstrates that a UWB based target tracking approach functions in an indoor environment where hundreds of UWB tags are present. However, the performance of such kind of ranging systems is strongly influenced by the propagation characteristics of UWB signal, in which the ranging errors in *LOS* and non-line-of-sight (*NLOS*) scenarios are dramatically different. Therefore, in-depth study of the impact of *LOS* and *NLOS* circumstances on the ranging accuracy is of significant practical importance for those mobile agent positioning/tracking related applications.

In recent years, many researchers have proposed reducing UWB ranging errors using Kalman filter (KF) based methods (including the KF variants based) and state discrimination based methods. The main idea in most KF based methods is to use the distance estimate at the previous time step as a priori to predict and use the distance measurement to update the ranging estimate at the current time step. In [8,9,10], the biased KF is used to process the ranging measurements, and the noise is assumed to follow the exponential distribution. However, when UWB sensors are deployed in structured and complex environments, the noise model of the system is often multi-modal, non-Gaussian, and biased, which lead to a significant degradation of ranging performance. In [11], an adaptive KF using colored noise is proposed to weaken the ranging error. The work proposed in [12] adjusts the gain of the KF dynamically according to the new interest, so as to weaken the ranging error and obtain a more accurate distance estimate. However, the parameters used in the method cannot be changed in an adaptive manner, and the performance is unsatisfactory in practice. Although the KF based methods can achieve real-time dynamic ranging, it has limited capability to model the highly uncertain ranging measurements well, in particular in the *NLOS* scenario. The use of KF based approaches is in addition constrained by the Gaussian assumption made in the kinematic model of the target.

The state discrimination based methods rely on the outcome of *LOS/NLOS* state identification to weaken the ranging error. These methods discriminate the *LOS* and *NLOS* states on the basis of the characteristic of the receive signal. This kind of method is straightforward and easy to implement, yet the performance highly depends on the accuracy of the *LOS/NLOS* hypothesis testing. In addition, the analysis of the signal channel for state identification using an analytical or machine learning based method inevitably introduces extra processing overhead, which is often significant, and, in many cases, the requirement of UWB hardware to provide received power envelope information. In [13,14], the kurtosis value of the RSS is used to achieve the state discrimination. A method based on RSS is proposed in [15] to identify *LOS* and *NLOS* paths, and it uses the least squares method to fit the ranging error and correct the ranging result. The method, however, does not show obvious reduction in the ranging error. The standard deviations of the measured samples and noise are adopted in [16] to identify the *LOS* and *NLOS* states. The approach is complicated and computationally demanding, which hinders the real-time implementation of the method. In recent years, there are machine learning approaches proposed for *LOS/NLOS* identification and error mitigation, e.g., support vector machine based in [17], relevance vector machine based in [18], and Gaussian processes based in [19]. The performance of these methods is, however, sensitive to the chosen kernel function, features extracted from received waveforms, tuning of parameters in the training process, etc.

Most existing state discrimination based approaches only produce deterministic and binary identification results, i.e., either *LOS* or *NLOS*, which are unable to provide further information regarding the significance of *NLOS* effect subject to obstruction materials and to treat it accordingly. The recent work in [20] is one of the first of its kind being able to consider multiple *NLOS* scenarios. It is, however, achieved based on received signal waveforms information, which requires UWB hardware support and the extraction of classification features. In addition, the channel identification result of the work is deterministic, which leads to unfavorable ranging results once the identification outcome could not precisely reflect the true *NLOS* scenario, especially during the transition of obstruction scenarios in the real world.

This paper proposes a Bayesian filtering based UWB ranging error weakening method, which can well model the nonparametric characteristics of random noise within a probabilistic framework, reduce ranging error in a typical indoor environment, and achieve real-time ranging. Statistical UWB sensor models are built based on real measurement data including the received signal strength (RSS) and the measured distance based on time of arrival (TOA) under *LOS* and multiple *NLOS* scenarios in an indoor environment. In particular, the RSS information conditional on measured range models the UWB signal propagation characteristics under the influence of different obstruction conditions and materials, which correspond to different magnitudes of *NLOS* effect. The approach probabilistically fuses the two types of measurements to yield more accurate ranging results yet at no additional cost, as the RSS information naturally comes with the distance measurements in UWB communication. Experiments were conducted for the proposed approach as (1) a standalone Bayesian error mitigation method under *LOS*, *NLOS*, and mixed propagation environments and (2) a measurement component integrated within another Bayesian state inference framework for mobile robot tracking. The experiment results show that the proposed ranging error mitigation approach can effectively weaken the peer-to-peer (P2P) ranging error, improve the tracking accuracy, and operate in real time.

The remainder of the paper is organized as follows. In Section 2, we briefly introduce the background on UWB based ranging and the environment of *LOS* and *NLOS*; The detailed principle of the proposed probabilistic UWB ranging error mitigation method and the Bayesian formulation are presented in Section 3; Experimental results are given and analyzed in Section 4; Finally, the conclusions and future work are given in Section 5.

## 2. Background on UWB Based Ranging

UWB technology mainly has two types of methods for P2P ranging, namely the RSS based and TOA based. There is another kind of approach known as time difference of arrival (TDOA) based, which is also exploiting time of flight principle. Therefore, TDOA methods are put into the TOA category for discussion in the paper. RSS based ranging requires the establishment of an accurate signal propagation model, which is not trivial in practice due to signal reflection, multi-path effects, and other clutter effects. The RSS based method is therefore usually used for rough ranging.

The TOA based method calculates the P2P distance by using the flight time of radio wave between the transmitter and receiver. Given Δt as the one-way signal flight time and *c* as the speed of radio wave, the distance between two nodes can be computed as d=cΔt. The TOA based method produces much higher accuracy of ranging than that of the RSS based approach. Nevertheless, the performance of the TOA based method is prone to the influence of obstacles between two nodes, especially metallic ones. For this reason, the TOA based method in UWB ranging has to consider two scenarios, namely *LOS* and *NLOS*, which are discussed in detail as follows.

### 2.1. LOS Scenario

The *LOS* scenario means that there is no obstruction between two communicating nodes. Generally, the ranging error in *LOS* scenario is small, mainly caused by timing error, multi-path effect, and antenna placement [15]. In some related work where high precision ranging is not required, e.g., in [21], the measured range obtained under the *LOS* scenario is considered as true distance. Nevertheless, precise P2P range information is desired in many applications—for instance, accurate tracking of mobile robots in indoor environments.

### 2.2. NLOS Scenario

In the *NLOS* scenario, the direct path between two communication nodes is blocked by obstacles, and the UWB radio signal can only reach the receiver node by means of reflection, refraction, and penetrating the obstacles. This is referred to as *NLOS* signal propagation in the literature, in which the measured RSS and distance based on TOA calculation cannot correctly reflect the true distance between two nodes. The random noise as a result becomes the main source of error in UWB ranging. The localization/tracking accuracy of mobile robots is significantly degraded or even not achievable given the considerable *NLOS* ranging error.

## 3. Probabilistic Ranging Error Mitigation

### 3.1. Building UWB Ranging Sensor Models

There are two probabilistic sensor models built and used in the proposed approach, which are

1. TOA model $P\left(z^{d}|d,s\right)$, where $s\in \left\{LOS,NLOS_{W},NLOS_{M}\right\}$ represents the communication obstruction and material scenario, *d* is the true Euclidean distance between two UWB communication nodes, and $z^{d}$ denotes measured distance through TOA.
2. RSS model $P\left(z^{rss}|z^{d},s\right)$, where *s* has been previously defined; $z^{rss}$ represents the measured received signal strength (RSS).

**Figure 1:**
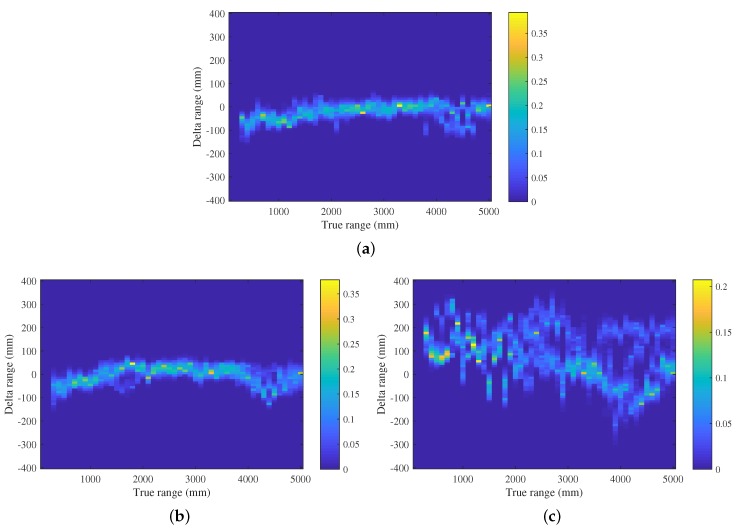
The time of arrival (TOA) sensor model built for different ultra-wideband (UWB) signal propagation cases, i.e., (**a**) line-of-sight (*LOS*); (**b**) non-line-of-sight with wood obstruction ($NLOS_{W}$); and (**c**) non-line-of-sight with metal obstruction ($NLOS_{M}$) cases using 132,651, 196,063, and 200,659 measurement samples, respectively.

To build the TOA model in practice, a large amount of range measurement samples are collected at a set of $N_{d}$ discrete true inter-node Euclidean distances $d\in \left\{d_{1},d_{2},\cdots ,d_{N_{d}}\right\}$, under *LOS* and *NLOS* scenarios. Note that here the NLOS scenario is further divided into two cases depending on the material of the obstacle, i.e., $NLOS_{W}$ for wood and $NLOS_{M}$ for metal. Each measurement of distance $z^{d}$ falls into one of $N_{zd}$ equal-sized bins. The TOA model is then constructed based on histograms of the measured range samples given each true distance. The model $P\left(z^{d}|d,s\right)$ built for each scenario is represented in the form of an $N_{zd}\times N_{d}$ matrix, where probability masses in each column are normalized so that they sum to unity. Figure 1 illustrates an example of TOA model built with 132,651, 196,063, and 200,659 samples for *LOS*, $NLOS_{W}$, and $NLOS_{M}$ scenarios, respectively. For a better visualization result, the TOA model is illustrated with $\Delta d=z^{d}-d$ on the *y*-axis in Figure 1. One can easily extend and well generalize the model by repeating the same process for more comprehensive communication environments and a larger group of obstacle types, in which case, we have a more general form of

(1) $$s\in \left\{LOS,NLOS_{1},NLOS_{2},\cdots ,NLOS_{N_{s}-1}\right\},$$

where $N_{s}$ represents the total number of propagation and obstruction material scenarios.

It can be seen from Figure 1a that the magnitude of ranging noise is generally stable and independent from distance in the *LOS* scenario. The noise is slightly biased by 50 mm for distances smaller than 1500 mm, and between 4000 and 5000 mm. The bias is found to be similar in the $NLOS_{W}$ scenario in Figure 1b. In the $NLOS_{M}$ case, as shown in Figure 1c, however, both the bias and magnitude of noise significantly increase. The bias itself also experiences dramatic fluctuation at all ranges tested.

Following the construction of the TOA model, the RSS model is constructed by statistically analyzing RSS samples associated with measured distances under *LOS* and *NLOS* scenarios. The same set of $N_{zd}$ bins used for measured distances in the TOA model is adopted. For each distance bin, those RSS samples with their associated measured distances falling within the bin are collected, and a histogram is generated by fitting them into $N_{zr}$ equal-sized RSS bins. This eventually produces the RSS model $P\left(z^{rss}|z^{d},s\right)$ in the form of an $N_{zr}\times N_{zd}$ matrix for each scenario. The elements in the matrices represent probability masses, which are normalized along the column direction.

**Figure 2:**
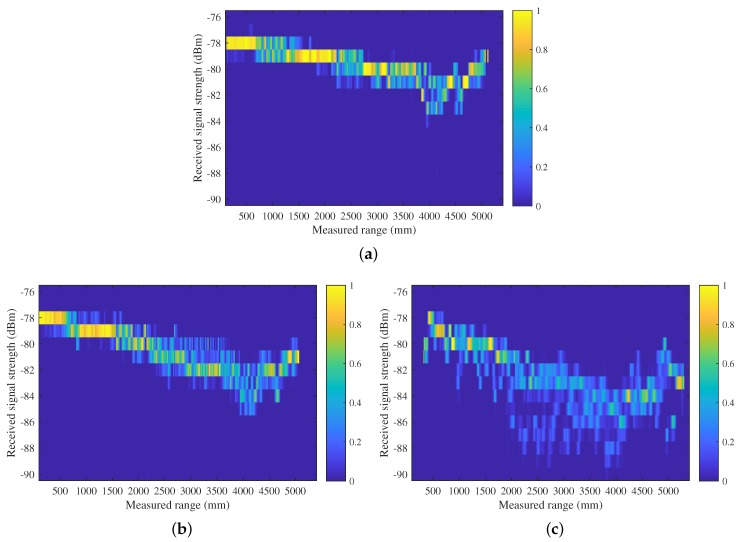
The received signal strength (RSS) sensor model built for (**a**) *LOS*; (**b**) $NLOS_{W}$; and (**c**) $NLOS_{M}$ UWB signal propagation cases based on 132,651, 196,063, and 200,659 measurement samples, respectively.

Figure 2 illustrates the RSS model in *LOS* and *NLOS* scenarios. Overall, the RSS value has a negative correlation with measured distance. Nevertheless, a slight increase of RSS value is observed for distances further than 4500 mm, which can be explained by constructive interference in multipath propagation of radio wave in the given testing setup and environment. As shown in Figure 2c, the attenuation rate and noise level of RSS in the $NLOS_{M}$ scenario are the greatest, followed by those in an $NLOS_{W}$ scenario in Figure 2b, and *LOS* scenario in Figure 2a.

It can be concluded from the models that the existence of obstacles between two communication nodes brings a detrimental effect on P2P ranging accuracy. In addition, conductive obstacles, for instance, those made of metal material, can cause a very high offset in UWB ranging.

### 3.2. Bayesian Filtering Formulation for Ranging Error Mitigation

The constructed probabilistic UWB sensor models can be used directly in a Bayesian filtering framework for reasoning the inter-node distance *d* and communication obstruction scenario *s* between the two UWB nodes. Overall, each iteration of the filtering can be divided into two steps.

#### 3.2.1. Prediction

(2) $$P\left(s_{t}|Z_{t-1}\right)=\sum_{s_{t-1}}P\left(s_{t}|s_{t-1}\right)P\left(s_{t-1}|Z_{t-1}\right),$$

where $P\left(s_{t}|s_{t-1}\right)$ is the transition function of *s* from time t−1 to *t*, $Z_{t-1}=\left\{z_{1}^{d},z_{1}^{rss},z_{2}^{d},z_{2}^{rss},\cdots ,z_{t-1}^{d},z_{t-1}^{rss}\right\}$ is a collection of historical measurements up to t−1, $P\left(s_{t-1}|Z_{t-1}\right)$ is the prior state, and $P\left(s_{t}|Z_{t-1}\right)$ is the prediction state. Equation (2) is based on the assumption that the transition of state *s* is a Markov process, i.e., the current state $s_{t}$ only depends on the previous state $s_{t-1}$.

As a simple example of the state transition function, $P\left(s_{t}|s_{t-1}\right)$ can be defined that the state at time t−1 has a probability of 0≤α≤1 to remain in the current state, while an equal chance to switch to either of other two states at *t*. In Equation (2), the prior state can be decomposed to

(3) $$P\left(s_{t-1}|Z_{t-1}\right)={\left[\begin{array}{ccc} P\left(s_{t-1}=LOS|Z_{t-1}\right) & P\left(s_{t-1}=NLOS_{W}|Z_{t-1}\right) & P\left(s_{t-1}=NLOS_{M}|Z_{t-1}\right) \end{array}\right]}^{T}.$$

Then, the state prediction process can be represented as an equation of matrix multiplication written as

(4) $$P\left(s_{t}|Z_{t-1}\right)=\left[\begin{array}{ccc} \alpha & \frac{1-\alpha }{2} & \frac{1-\alpha }{2}\\ \frac{1-\alpha }{2} & \alpha & \frac{1-\alpha }{2}\\ \frac{1-\alpha }{2} & \frac{1-\alpha }{2} & \alpha \end{array}\right]P\left(s_{t-1}|Z_{t-1}\right).$$

It should be noted that, by assuming constant probabilities of *s* staying in the current state and switching to one of the other propagation and obstruction scenarios at a new time step, the state transition function is independent from the current location of the target or other states. It is therefore not required to have the layout of the environment or locations of obstructions as a priori information.

#### 3.2.2. Update

Given a pair of measured distance and RSS at time *t*, i.e., $z_{t}=\left\{z_{t}^{d},z_{t}^{rss}\right\}$, we are able to obtain the likelihood function of inter-node distance given the measurements through the UWB sensor models. The joint likelihood function given the pair of measurements can be written as

(5) $$P\left(z_{t}^{rss},z_{t}^{d}|d,s_{t}\right)=P\left(z^{rss}=z_{t}^{rss}|z^{d}=z_{t}^{d},s_{t}\right)P\left(z^{d}=z_{t}^{d}|d,s_{t}\right),$$

where $P\left(z^{rss}=z_{t}^{rss}|z^{d}=z_{t}^{d},s_{t}\right)$ and $P\left(z^{d}=z_{t}^{d}|d,s_{t}\right)$ are likelihood functions given the RSS and TOA sensor models, respectively.

Then, the update step of the Bayesian filtering can be written as

(6) $$P\left(d,s_{t}|Z_{t}\right)\propto P\left(z_{t}^{d},z_{t}^{rss}|d,s_{t}\right)P\left(s_{t}|Z_{t-1}\right).$$

From the posterior state $P\left(d,s_{t}|Z_{t}\right)$, we can further extract estimates of $s_{t}$ and distance produced at time *t*. The estimate of $s_{t}$ is calculated as

(7) $$P\left(s_{t}|Z_{t}\right)=\sum_{i=1}^{N_{d}}P\left(d=d_{i},s_{t}|Z_{t}\right),$$

which also serves as the prior in the Bayesian filtering at the next time step.

The marginal probabilities $P_{LOS}=P\left(s_{t}=LOS|Z_{t}\right)$, $P_{NLOS_{W}}=P\left(s_{t}=NLOS_{W}|Z_{t}\right)$, and $P_{NLOS_{M}}=P\left(s_{t}=NLOS_{M}|Z_{t}\right)$ describe the chances that the UWB communication of two nodes takes place in the corresponding communication obstruction scenarios.

The probability mass at the *i*th distance bin can be obtained by marginalizing $s_{t}$ away, i.e.,

(8) $$P_{d}\left(i\right)=\sum_{s_{t}}P\left(d=d_{i},s_{t}|Z_{t}\right).$$

Lastly, the mean of distance estimate can be computed as

(9) $$\bar{d}=\sum_{i=1}^{N_{d}}d_{i}P_{d}\left(i\right).$$

Please refer to Section 4.2 for experimental validation of the probabilistic UWB sensor models and the Bayesian filtering framework used as a standalone ranging error mitigation approach.

### 3.3. Bayesian Filtering Formulation for Mobile Target Tracking in a Wireless Sensor Network

The proposed ranging error mitigation method can be incorporated as a component within a mobile target Bayesian tracking framework in a wireless sensor network. Define a joint state vector $X_{t}$ at time *t* that considers the state of a mobile target and the obstruction scenarios between the target and each of $N_{ref}$ reference points that form the wireless sensor network. It can be written as

(10) $$X_{t}={\left[\begin{array}{cc} {\left(x_{t}\right)}^{T} & {\left(s_{t}\right)}^{T} \end{array}\right]}^{T},$$

where $s_{t}={\left[\begin{array}{cccc} s_{t}^{1} & s_{t}^{2} & \cdots & s_{t}^{N_{ref}} \end{array}\right]}^{T}$ and $s_{t}^{i},\;i=1,2,\cdots ,N_{ref}$ denotes the obstruction scenario to the corresponding reference point *i* at time *t*. In Equation (10), $x_{t}$ represents the state vector of the target, which varies depending on the applications. An example of $x_{t}$ for mobile robot tracking in 2D space can be written as

(11) $$x_{t}={\left[\begin{array}{ccccc} x_{t} & y_{t} & \theta_{t} & v_{t} & \omega_{t} \end{array}\right]}^{T},$$

where $x_{t}$, $y_{t}$, $\theta_{t}$ are pose variables representing *x* and *y* position coordinates and orientation, respectively, and $v_{t}$ and $\omega_{t}$ are kinematic parameters describing instantaneous linear and angular velocities, respectively, at time *t*.

#### 3.3.1. Prediction

(12) $$P\left(X_{t}|Z_{t-1}^{*}\right)=\int P\left(X_{t}|X_{t-1}\right)P\left(X_{t-1}|Z_{t-1}^{*}\right)dX_{t-1},$$

where $P\left(X_{t}|X_{t-1}\right)$ is the joint state transition function from time t−1 to *t*, $P\left(X_{t-1}|Z_{t-1}^{*}\right)$ is the prior joint state, $P\left(X_{t}|Z_{t-1}^{*}\right)$ is the prediction, $Z_{t-1}^{*}=\left\{z_{1}^{*},z_{2}^{*},\cdots ,z_{t-1}^{*}\right\}$ is a collection of historical sensor measurements up to t−1. The transition function here includes both the prediction of obstruction scenarios, and the prediction of the pose and kinematic variables. We make the following assumptions:

1. The target moves independently in the field, which means the current target state is only dependent on the last state, i.e., the Markov assumption. This implies that $P\left(x_{0:t}\right)=\left(\prod_{k=1}^{t}P\left(x_{k}|x_{k-1}\right)\right)P\left(x_{0}\right)$
2. The transitions of obstruction scenario between the target and each reference point are independent from one another, which means $P\left(s_{t}|s_{t-1},x_{t-1}\right)=\prod_{i=1}^{N_{ref}}P\left(s_{t}^{i}|s_{t-1}^{i},x_{t-1}\right)$, where $P\left(s_{t}^{i}|s_{t-1}^{i},x_{t-1}\right)$ is the transition function of $s^{i}$ that is conditional on the previous state of the target

With the aforementioned assumptions made, the transition function of the joint state can be further decomposed to

(13) $$P\left(X_{t}|X_{t-1}\right)=\left(\prod_{i=1}^{N_{ref}}P\left(s_{t}^{i}|s_{t-1}^{i},x_{t-1}\right)\right)P\left(x_{t}|x_{t-1}\right),$$

where $\left(x_{t}|x_{t-1}\right)$ refers to the kinematic model of the mobile target.

In many applications where no correlation is assumed between the obstruction scenarios and target state, we simply have $P\left(s_{t}^{i}|s_{t-1}^{i},x_{t-1}\right)=P\left(s_{t}^{i}|s_{t-1}^{i}\right)$.

#### 3.3.2. Update

The Bayesian update step is conducted by fusing all sensor measurements available at time *t*

(14) $$z_{t}^{*}=\left\{z_{t}^{x},z_{t}^{1},z_{t}^{2},\cdots ,z_{t}^{N_{ref}}\right\},$$

where $z_{t}^{i},\;i=1,2,\cdots ,N_{ref}$ is the pair of distance and RSS measured by the UWB sensor of reference point *i*, and $z_{t}^{x}$ refers to the set of other sensor measurements only conditional on the target state. Please note that $z_{t}^{x}$ is optional, as using the UWB measurements is usually sufficient to constrain the target state estimation.

The update step of the Bayesian filtering is written as

(15) $$P\left(X_{t}|Z_{t}^{*}\right)\propto \left(\prod_{i=1}^{N_{ref}}P\left(z_{t}^{i}|d_{t}^{i},s_{t}^{i}\right)\right)P\left(z_{t}^{x}|x_{t}\right)P\left(X_{t}|Z_{t-1}^{*}\right),$$

where $P\left(z_{t}^{i}|d_{t}^{i},s_{t}^{i}\right)$ is the joint likelihood function given the UWB measurements, as previously defined in Equation (5), $d_{t}^{i}=\sqrt{{\left(x_{t}-x^{i}\right)}^{2}+{\left(y_{t}-y^{i}\right)}^{2}}$ is the Euclidean distance between the target’s predicted position $x_{t}$, $y_{t}$ and position $x^{i}$, $y^{i}$ of reference point *i*.

The filtering runs the prediction step in Equation (12) and the update step in Equation (15) iteratively. The marginal probability distribution of the target’s state at time *t* can be acquired by integrating Equation (15) with respect to the obstruction states in the joint state vector.

The experimental results of tracking a mobile robot using the UWB ranging error mitigation method as a component in the Bayesian filtering framework formulated in this section are available in Section 4.3.

## 4. Experimental Results and Analysis

### 4.1. Experimental Platform

**Figure 3:**
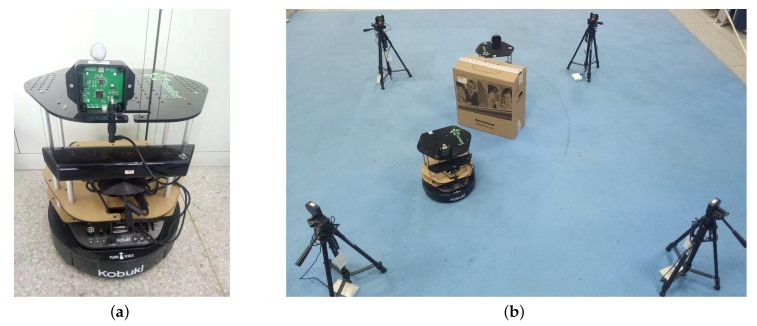
Inter-robot ranging experiment setup. (**a**) shows a TurtleBot2 robot with a UWB module; (**b**) depicts the overall experiment setup. The carton shown in (**b**) was used as a container for wood or metal obstacles.

**Figure 4:**
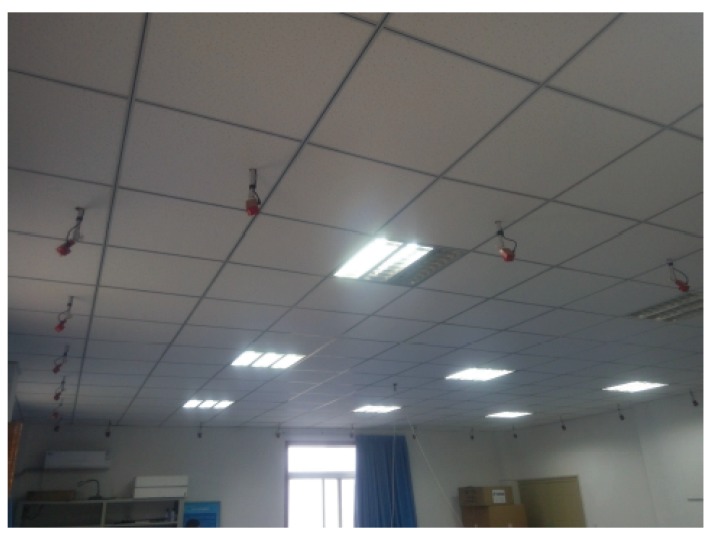
OptiTrack motion capture system.

To validate the proposed approach, we built a UWB sensor ranging platform shown in Figure 3a in the robot operating system (ROS) environment. As demonstrated in Figure 3b, a few Pozyx UWB ranging modules and TurtleBot2 mobile robots were employed in two experiments conducted, with their real-time ground truth positions acquired by the OptiTrack motion capture system shown in Figure 4. The OptiTrack system is composed of 24 high-definition cameras, covering the region of interest for the experiments. Through calibration, the OptiTrack motion capture system is providing 3D triangulation with precision on the order of $10^{-3}$ m, which is sufficient to be used as ground truth in the experiments. The UWB modules were mounted at a fixed height of 0.5 m. The raw RSS and range measurements were provided by the Pozyx SDK.

### 4.2. Inter-Robot Ranging Experiment Results

In the first set of experiments, two mobile robots equipped with UWB ranging modules autonomously navigated using on-board red green blue and depth (RGB-D) sensors to opposite directions in an indoor environment, which results in variation of inter-robot range over time, as illustrated in Figure 3b. Four subcases, namely *LOS*, $NLOS_{W}$, $NLOS_{M}$, and a mixture of them, were tested by placing different kinds of obstacles between two mobile robots, or not placing any obstacle. The mixture subcase is the most complicated among the four subcases tested. It created a mixed P2P communication scenario that switched between *LOS*, $NLOS_{W}$, and $NLOS_{M}$ along with the relative movement of mobile robots.

**Figure 5:**
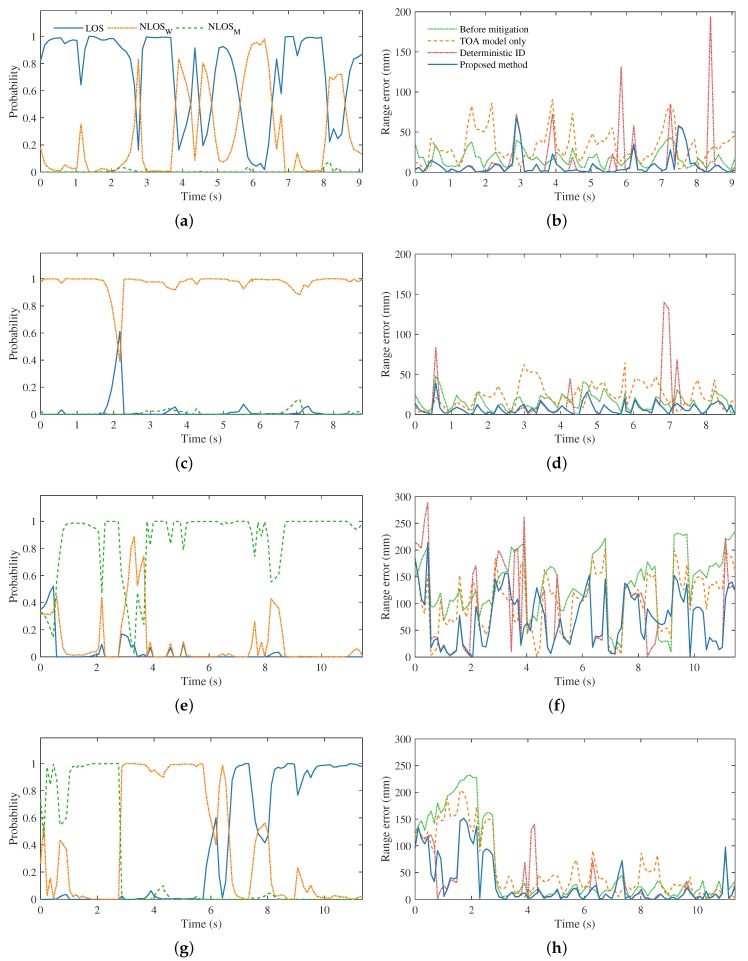
Experiment results of the inter-robot ranging. The obstacle type identification results of *LOS*, $NLOS_{W}$, $NLOS_{M}$ and the mixture subcases are shown in (**a**,**c**,**e**,**g**), respectively. The ranging error results of the corresponding subcases are presented, respectively, in (**b**,**d**,**f**,**h**).

During the experiment, measurements from UWB modules were obtained at 8.8 Hz. The range estimates using the propose method were compared with the ground truth values from the OptiTrack system at 50 Hz. The parameter α used in the state transition function $P\left(s_{t}|s_{t-1}\right)$ is set to 0.95, and $P_{LOS}$, $P_{NLOS_{W}}$, and $P_{NLOS_{M}}$ are initialized with equal probabilities. The ranging errors before and after the use of the proposed approach are shown in Figure 5. It can be seen from Figure 5 that, in all tested subcases, the proposed approach overall can effectively reduce the error in P2P ranging. The obstacle type identification in the every subcase is accurate for the majority of time. In the mixture subcase, as depicted in Figure 5g, the identification result is clearly observed to shift from $NLOS_{M}$ to $NLOS_{W}$ and lastly *LOS*, which is expected.

In terms of error mitigation, the proposed approach manages to reduce the ranging error significantly in every subcase. In particular, the calculated range estimates in *LOS* and $NLOS_{W}$ subcases are close to ground truth distance throughout the experiments, as shown in Figure 5b,d respectively. In the $NLOS_{M}$ subcase, the raw range measurements substantially deviate from the true values, reaching an error as high as 200 mm, which can be seen in Figure 5f. The ranging errors in this subcase are significantly reduced after the proposed mitigation method is applied.

Besides, in these figures, the results of the proposed method are compared with that produced by using the built TOA model only, which means we do not identify the obstacle type in the use of RSS model and assume $P_{LOS}=P_{NLOS_{W}}=P_{NLOS_{M}}=\frac{1}{3}$ all the time. It can be concluded that, without the knowledge of obstacle identification, the ranging errors after only a TOA model is used are even larger than that before mitigation in *LOS* and $NLOS_{W}$ subcases. The proposed approach is also compared with the deterministic ID method, where the obstacle scenario with the highest identification score is considered in error mitigation, which represents the case in most of existing state identification based error mitigation approaches. It can be seen that the deterministic ID method also reduces the ranging error for the majority of time, yet its performance is inferior to that of the proposed approach. The ranging results presented in Figure 5 are also compared in Table 1 in terms of mean ranging error in every subcase.

It is observed in the *LOS* subcase, as shown in Figure 5a, the identification result with the highest probability alternating a few times between *LOS* and $NLOS_{W}$ scenarios throughout the experiment period, due to the fact that the UWB sensor characteristics in *LOS* and $NLOS_{W}$ environments are very close. Similarly, the identification result switched to $NLOS_{W}$ in the $NLOS_{M}$ subcase for a short period of time, as demonstrated in Figure 5e. However, the proposed approach still weakened the ranging error during these periods, mainly because it is able to reason and combine the multiple identification scenarios probabilistically within a Bayesian filtering framework. On the other hand, the deterministic ID method is sensitive to the correctness of the obstruction identification result. Some spikes in range error are found in each subcase when the identification is ambiguous. The proposed approach therefore shows its robustness against inaccuracy in identification of UWB propagation and obstruction material scenarios.

It is concluded from the above experimental results that the proposed probabilistic ranging method can effectively model the sensor characteristics and mitigate the UWB ranging error in the presence of different degrees of *NLOS* effect. The method also has excellent real-time computational performance by processing a pair of measurements within 0.07 ms on a computer with Matlab 2017b (MathWorks, Natick, MA, USA) and Intel Xeon E3-1230 v3 CPU (Santa Clara, CA, USA).

### 4.3. Mobile Robot Tracking Experiment Results

**Figure 6:**
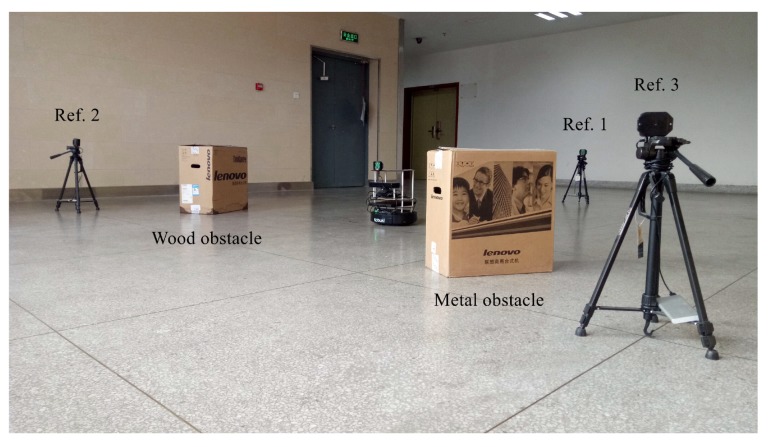
Mobile robot tracking experiment setup. The cartons were used as containers for wood and metal obstacles for the convenience of placement.

**Figure 7:**
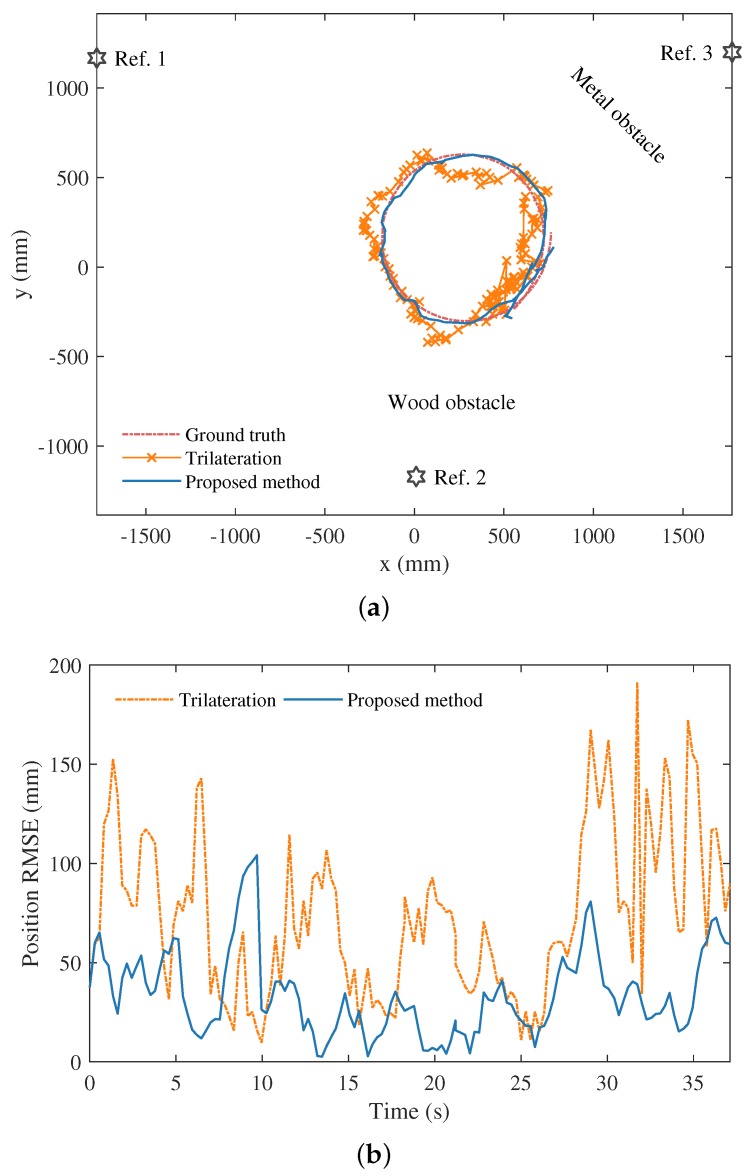
Experiment results in mobile target tracking. (**a**) depicts the three UWB reference points deployed in the experiment. In addition, the estimated trajectory of the mobile robot using the proposed method, trajectory based on trilateration of raw range measurements, and their ground truth are shown in (**a**). The comparison of the position root mean squared errors (RMSEs) of using the proposed approach and the trilateration is presented in (**b**).

The proposed error mitigation approach has also been validated in a mobile robot tracking experiment as a part of the Bayesian tracking framework as described in Section 3.3. The experiment was setup with a wireless sensor network of three UWB reference points and a single mobile robot moving in an indoor environment, as illustrated in Figure 6. There was no obstacle between the robot and the reference point 1. Wood and metal obstacles were placed between the robot and reference point 2 and 3, respectively, which are shown in Figure 6 and Figure 7a.

In the experiment, the mobile robot completed a revolution of circular motion over a period of 30 s, during which raw UWB measurements between the robot and three reference points were obtained at a frequency of approximately 3 Hz. The proposed approach is integrated within a bootstrap particle filter, which is the preferred choice given the nonparametric nature of the UWB sensor models built in the paper. The quantity of particles used in the filter is set to 1000. The parameter α for probabilistic transition of *s* is set to 0.90 in the experiment. A constant turn rate and velocity (CTRV) model is assumed to describe the kinematics of the mobile robot. Please note that the mobile robot is tracked by solely relying on the noisy RSS and relative ranging information in UWB communication. No knowledge of robot egocentric inertial or odometry data is used for constraining the robot state estimates. Lastly, the real-time 2D position estimates of the particle filter were compared with the ground truth values acquired from the OptiTrack system.

It can be seen from Figure 7a that the robot trajectory estimated by the proposed approach is close to the ground truth. The estimated trajectory of the robot is also compared with the noisy trajectory formed by 2D trilateration using the UWB ranging data before error mitigation in Figure 7a. To quantitatively assess the positioning performance, the root mean squared error (RMSE) of position is employed as the evaluation metric, which is computed at time step *t* by

(16) $$e_{t}^{pos}=\sqrt{\frac{{\left({\hat{x}}_{t}-x_{t}^{\prime }\right)}^{2}+{\left({\hat{y}}_{t}-y_{t}^{\prime }\right)}^{2}}{2}},$$

where ${\hat{x}}_{t}$ and ${\hat{y}}_{t}$ are either means of position estimates produced by the particle filter at time *t*, or the 2D trilateration results using the measured ranges at the time step, $x_{t}^{\prime }$ and $y_{t}^{\prime }$ are their ground truth values, respectively.

In Figure 7b, the RMSE of the estimated trajectory using the proposed approach and particle filtering is overall found to be substantially smaller than that before error mitigation during the experiment, with the mean RMSE reduced from 73.24 to 33.87 mm. Nevertheless, an exception is observed for a short period, i.e., from time 8 to 10 s. This was caused by outlier measurements, which means those lying beyond the prediction of the UWB sensor models built for the experiment. The issue can be overcome by generalizing the sensor models with more samples and with those collected under more diverse scenarios.

On the same computer platform used in Section 4.2, the processing of the proposed mitigation approach related part in each iteration of the particle filter takes no more than 1.5 ms given the 1000 particles, which can meet the real-time processing requirement.

The tracking results were obtained with only three reference sensor points in the experiment. Overall, the accuracy of positioning/tracking can be improved by placing more reference points. It also depends on the geometric constellation of the references in the environment. Nevertheless, with a greater number of references, the contribution from each additional reference point decreases. The positioning is sufficiently accurate when the number of reference nodes reaches a certain amount. It then becomes a trade-off between the further gain in positioning accuracy and the additional computational cost of the mitigation and positioning/tracking approaches. One of the primitive metrics to quantify the accuracy of trilateration in 2D space is the horizontal dilution of precision (HDOP), which can be used as a general guide to choosing the optimal quantity and deployment of reference points in the environment for positioning or tracking.

Given a total of $N_{ref}$ reference points that are used to position the target, we first build up a matrix $A_{t}$ as

(17) $$A_{t}=\left[\begin{array}{cc} \frac{\left(x_{t}^{\prime }-x^{1}\right)}{r_{t}^{1}} & \frac{\left(y_{t}^{\prime }-y^{1}\right)}{r_{t}^{1}}\\ \frac{\left(x_{t}^{\prime }-x^{2}\right)}{r_{t}^{2}} & \frac{\left(y_{t}^{\prime }-y^{2}\right)}{r_{t}^{2}}\\ \vdots & \vdots \\ \frac{\left(x_{t}^{\prime }-x^{N_{ref}}\right)}{r_{t}^{N_{ref}}} & \frac{\left(y_{t}^{\prime }-y^{N_{ref}}\right)}{r_{t}^{N_{ref}}} \end{array}\right],$$

where $r_{t}^{i}=\sqrt{{\left(x_{t}^{\prime }-x^{i}\right)}^{2}+{\left(y_{t}^{\prime }-y^{i}\right)}^{2}},\;i=1,2,\cdots ,N_{ref}$ is the Euclidean distance between true position of the target at time *t* and the position of reference point *i*. Then, we formulate another matrix $Q_{t}$ as

(18) $$Q_{t}={\left(A_{t}^{T}A_{t}\right)}^{-1}.$$

Lastly, the HDOP at time *t* is calculated as

(19) $$HDOP_{t}=\sqrt{\sigma_{x}^{2}+\sigma_{y}^{2}},$$

where $\sigma_{x}$ and $\sigma_{y}$ are obtained by reformulating Equation (18) in the form of $Q_{t}=\left[\begin{array}{cc} \sigma_{x}^{2} & \sigma_{xy}\\ \sigma_{xy} & \sigma_{y}^{2} \end{array}\right]$.

### 4.4. Discussion

As seen and discussed in Section 3, the formulation of the proposed ranging error mitigation approach does not pose a theoretical upper bound on the number of obstruction material scenarios it could manage simultaneously in an environment. Three propagation and obstruction material scenarios, i.e., *LOS*, *NLOS* with wood obstruction, and *NLOS* with metal obstruction, are chosen and investigated in the proposed approach and experiments mainly because these are representative scenarios in an indoor environment, corresponding to no *NLOS* effect, mild *NLOS* effect and serious *NLOS* effect, respectively. These are suitable examples for the purpose of demonstrating the rationale and strengths of the proposed approach. The proposed approach can be implemented in an environment of different signal propagation characteristics. It can also be easily extended to incorporate more obstruction material cases, e.g., human body and concrete wall, to work in a more complex environment and/or to produce finer mitigation results. Given a new target environment, the typical obstruction materials are first studied and grouped into a few categories. The same process of building both TOA and RSS sensor models presented in Section 3.1 needs to be repeated for each of the obstruction material scenarios under consideration.

Admittedly, considering more obstacle material scenarios in a more complicated environment would increase the computational cost of the proposed method. It requires a higher overall cost for building TOA and RSS models for every obstruction material, which however is one-off, and carried out offline. In addition, it causes an increased computational cost for the online ranging and tracking algorithms due to more sensor models of obstruction scenarios considered in every iteration of Bayesian filtering. Nevertheless, with the rest of parameters kept the same, the time complexity of the proposed approach is a linear function of the number of propagation and obstruction material scenarios, i.e., $O\left(N_{s}\right)$, where $N_{s}$ has been defined in Equation (1). This level of complexity is considered acceptable. The communication cost is independent from the quantity of obstruction materials considered. This is because only local information, i.e., TOA based range measurement and RSS data, is used in error mitigation. No extra cost is required from the communication perspective for ranging error mitigation.

## 5. Conclusions and Future Work

The paper proposes a novel Bayesian inference based ranging error mitigation approach. Nonparametric probabilistic UWB sensor models built using RSS and TOA data were constructed in a typical indoor environments with different signal propagation and obstruction scenarios, namely, *LOS*, *NLOS* with wood obstacle, and *NLOS* with metal obstacles. The approach probabilistically combines the two kinds of information in UWB communication and comes up with multiple channel hypotheses following a Bayesian filtering formulation. Experiments were conducted to validate the proposed approach under two configurations, i.e., (1) used as a standalone probabilistic method for P2P ranging error mitigation using UWB sensors; and (2) used as a component within a more complicated Bayesian estimation framework for mobile target tracking in a UWB sensor network. It is also demonstrated that the proposed method requires a low computational cost, which allows real-time operation when implemented in actual systems.

The future work includes improvement of the UWB sensor models in more challenging environments and considers a broader variety of signal propagation scenarios and obstacle materials. In addition, the model can be improved in the future to utilize machine learning techniques—for instance, online learning of sensor properties to make it more robust against outliers and change of environments. Lastly, another application of the proposed approach in the future is to be incorporated in a cooperative tracking paradigm for multiple mobile robots.

## Figures and Tables

**Table 1:** Mean ranging errors in an inter-robot ranging experiment. The best result in each row is highlighted in **bold**.

Subcase	Before Mitigation (mm)	TOA Model Only (mm)	Deterministic ID (mm)	Proposed Method (mm)
*LOS*	16.90	32.63	15.24	**8.19**
$NLOS_{W}$	15.41	23.57	12.23	**7.42**
$NLOS_{M}$	135.53	104.83	85.40	**72.10**
Mixture	57.33	57.55	33.00	**28.92**

Abbreviations: TOA, time of arrival; *LOS*, line-of-sight; $NLOS_{W}$, non-line-of-sight with wood obstruction; $NLOS_{M}$, non-line-of-sight with metal obstruction.

## Abbreviations

The following abbreviations are used in this manuscript:

UWB	ultra-wideband
LOS	line-of-sight
NLOS	non-line-of-sight
RSS	received signal strength
TOA	time of arrival
GNSS	global navigation satellite system
KF	Kalman filter
P2P	peer-to-peer
TDOA	time difference of arrival
ROS	robot operating system
RGB-D	red green blue and depth
CTRV	constant turn rate and velocity
RMSE	root mean squared error
HDOP	horizontal dilution of precision
